# Transcription, Epigenetics and Ameliorative Strategies in Huntington’s Disease: a Genome-Wide Perspective

**DOI:** 10.1007/s12035-014-8715-8

**Published:** 2014-05-01

**Authors:** Luis M. Valor

**Affiliations:** Instituto de Neurociencias de Alicante, Universidad Miguel Hernández—Consejo Superior de Investigaciones Científicas, Av. Santiago Ramón y Cajal s/n, Sant Joan d’Alacant, 03550 Alicante, Spain

**Keywords:** Polyglutamine, Transcriptome, Epigenome, Microarray, Next-generation sequencing, Amelioration

## Abstract

Transcriptional dysregulation in Huntington’s disease (HD) is an early event that shapes the brain transcriptome by both the depletion and ectopic activation of gene products that eventually affect survival and neuronal functions. Disruption in the activity of gene expression regulators, such as transcription factors, chromatin-remodeling proteins, and noncoding RNAs, accounts for the expression changes observed in multiple animal and cellular models of HD and in samples from patients. Here, I review the recent advances in the study of HD transcriptional dysregulation and its causes to finally discuss the possible implications in ameliorative strategies from a genome-wide perspective. To date, the use of genome-wide approaches, predominantly based on microarray platforms, has been successful in providing an extensive catalog of differentially regulated genes, including biomarkers aimed at monitoring the progress of the pathology. Although still incipient, the introduction of combined next-generation sequencing techniques is enhancing our comprehension of the mechanisms underlying altered transcriptional dysregulation in HD by providing the first genomic landscapes associated with epigenetics and the occupancy of transcription factors. In addition, the use of genome-wide approaches is becoming more and more necessary to evaluate the efficacy and safety of ameliorative strategies and to identify novel mechanisms of amelioration that may help in the improvement of current preclinical therapeutics. Finally, the major conclusions obtained from HD transcriptomics studies have the potential to be extrapolated to other neurodegenerative disorders.

## Introduction

Huntington’s disease (HD) is the most common polyglutamine (polyQ) disorder, with a prevalence of 5–10 cases per 100,000 persons worldwide. In the classical variant, pathology onset occurs in mid adulthood (approximately 30–40 years old) and ends with the patient’s death, normally after 10–15 years. HD is characterized by personality changes and psychiatric symptoms (e.g., affective disorders, suicide tendency, mania, apathy, and schizophrenia-like symptoms), cognitive deficits (e.g., poor planning and judgment, attention problems, and motor skill learning deficits), motor impairment (e.g., chorea, gait abnormalities, bradykinesia, and rigidity), sleep disturbance, and weight loss [1]. The basal ganglia and, in particular, the striatum, wherein the GABAergic medium spiny neurons (MSN) are especially sensitive, generally show an early marked degeneration that extends to other brain areas in later stages of the disease [1].

Conversely to more common neurodegenerative disorders, such as Alzheimer’s (AD) and Parkinson’s (PD) diseases, the cause of HD for all the patients is known: an aberrant expansion of the polymorphic trinucleotide sequence CAG (in the range of 37–121 repeats) at the N-terminus of the huntingtin (Htt) protein. Whereas the number of repeats is strongly correlated with the age of disease onset, additional genetic variants may contribute to the differential manifestation of the cognitive and motor symptomatology among patients [2]. In any case, the mutation has two different consequences [3]: (i) a loss-of-function effect, as one allele is no longer available for the physiological roles of normal Htt such as vesicle trafficking and synaptic transmission, and (ii) a gain-of-function effect, characterized by the presence of a misfolded mutant Htt (mHtt) that interferes with multiple intracellular activities through aberrant interactions and accumulates in aggregates, mainly in the cell nucleus and neuropil. Overall, these combined effects alter several cellular processes, such as protein degradation, mitochondrial respiration and transcription among many others, eventually leading to neuronal malfunction and cell loss.

Initial reports during the 1980s and 1990s demonstrated selective altered expression of highly identifiable neuronal genes, such as neurotransmitter receptors and neuropeptides, first in patients’ brains and later in animal models [4–7]. The importance of transcriptional dysregulation in the pathology of HD was demonstrated by nuclear-restricted variants of mHtt transgenes that reproduced part of the HD symptomatology [8, 9]. With the advent of genome-wide approaches, transcriptomics was soon adopted to extend the catalog of affected transcripts in HD and to identify the most compromised cellular processes due to the detrimental supply of key components from the nucleus. However, the transcriptome is not limited to protein-coding genes, which represent less than 5 % of the genome, and new classes of noncoding RNAs are currently in the spotlight. The continuous development of transcriptomics technologies and concurrent bioinformatic tools is increasingly enlarging our global perspective of the transcriptional phenomenon. Currently, deep sequencing techniques coexist with newly improved array platforms to maximize coverage of the entire transcriptome, including noncoding genomic features and alternative isoforms, and to connect transcriptomics with other sources of genome-wide data related to DNA modification and the occupancy of DNA-binding proteins (histones and transcription factors). In this review, I discuss the role of transcriptional dysregulation in the pathology of HD and the postulated causes for this impairment, with special emphasis on the information generated by high-throughput techniques, as they have the potential to provide a nearly complete vision of the genome regulation in response to pathology and restorative strategies.

## Widespread Transcriptional Dysregulation in Huntington’s Disease

**Fig. 1:**
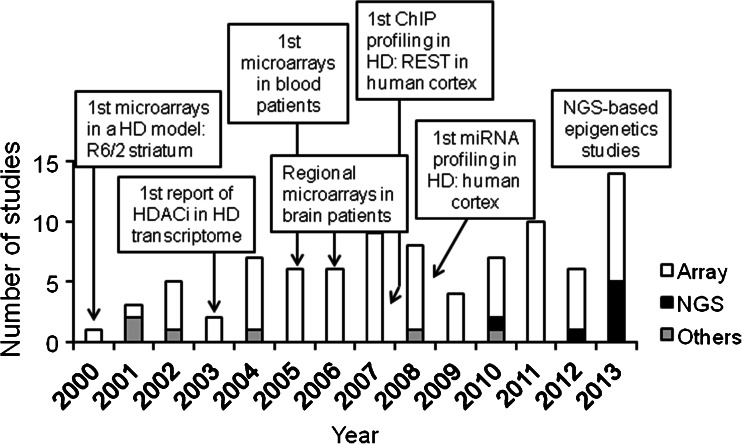
Summary of the gene profiling and other genome-wide studies in HD. The *graph* represents the number of studies per publication year with the most relevant milestones in the field of HD transcriptional dysregulation. Note that a few studies may contain more than one approach, categorized as “Array” (expression and ChIP-on-chip), “NGS” (next-generation sequencing, RNA-seq, ChIP-seq, sequencing of methylated DNA) and “Others” (PCR-based: differential display, adapter-tagged competitive PCR, panels). Reports with solely reanalysis (i.e., without generating data de novo) or chemically lesioned striatal models are not considered. References per year: 2000, [22]; 2001, [10, 15]; 2002: [12, 23, 24, 31, 65]; 2003, [173, 193]; 2004, [11]; 2005, [9, 25, 51, 135, 159, 194]; 2006, [26, 34, 36, 37, 157, 195]; 2007, [32, 38, 52, 54, 72, 110, 133, 154, 196]; 2008, [13, 27, 33, 39, 56, 103, 125, 155]; 2009, [35, 151, 152, 197]; 2010, [14, 16, 158, 165, 174, 192]; 2011, [18, 19, 30, 50, 57, 67, 119, 127, 156, 198]; 2012, [17, 66, 99, 111, 112, 181, 199]; 2013, [20, 21, 28, 29, 49, 53, 68, 113, 116, 124, 128, 180, 200]

A long list of gene expression datasets have been generated in less than 15 years of research on multiple HD animal and cellular models and, importantly, on postmortem material from patients (Fig. 1). The preferred system for the analysis of HD transcriptomics has been microarray technology, with some studies using alternative approaches, such as differential display transcription [10, 11], to capture novel changing genes not represented by the arrays at the time, or systematic qPCR-based assays [12–15], to selectively interrogate specific subsets of genes with high sensitivity as in the case of microRNAs (miRNAs), prior to the use of dedicated microarrays and deep sequencing technologies [16–19]. The results obtained using the most novel RNA-seq approach are consistent with microarray-based profiles [16, 20, 21], with the added benefit that they analyze the behavior of poorly characterized gene products, such as variants of mature miRNA or isomiRs [16].

An overview of the major conclusions derived from HD transcriptomics is given below.

### Transcriptional Dysregulation Is an Early and Progressive Event

Profiling studies in HD animal models usually explore a minimally symptomatic stage at the phenotypical and morphological level that, in contrast, show a substantially disrupted transcriptome that worsens during progression of the disease [17, 22–30]. Further demonstrations of the prematurity of transcriptional alterations have been provided by highly controlled in vitro preparations in which gene expression changes occur prior to cell loss, mHtt aggregation, or mitochondrial dysfunction [12, 15, 31–35]. Additionally, evidence for transcriptional alterations was provided by the laser capture microdissection (LCM) of intact neurons from early symptomatic brain samples, which yielded a transcriptional profile largely comparable to that obtained from whole homogenates [36], indicating that a significant component of the HD signature in tissues is not derived from neuronal loss.

However, transcriptional dysregulation is not too early on time [23, 33, 37, 38], and accumulation of events are seemingly needed (e.g., mHtt concentration and nuclear shuttling) to overtly affect regulatory mechanisms. In any case, the progression of gene expression and phenotype can be correlated, as demonstrated by the identification of genes associated with performance impairment in the rotarod task and in decreased exploratory and spontaneous activities [39].

### Transcriptional Dysregulation Affects Relevant Genes for Brain Function

Diverse processes are usually represented in HD transcriptional profiles: downregulation is generally associated with genes involved in signaling pathways (e.g., dependent on cyclic nucleotides and trophic factors), neurotransmitter receptor and ion channel functions (e.g., dopamine, serotonin, adenosine, GABA, and glutamate receptors), neuropeptides (e.g., proenkephalin and somatostatin), synaptic transmission (anchoring and vesicle components), and calcium binding and homeostasis, whereas upregulation is generally associated with genes related with RNA metabolism, protein folding, and stress markers. Expression of transcription and chromatin remodeling factors can be altered in either direction. A more exhaustive discussion of the biological implications of deregulated genes in the context of HD can be found in the excellent reviews by Cha [40] and Seredenina and Luthi-Carter [41]. Functional information can be obtained from transcriptomics studies that not only provides potential causative contributors for perturbed cellular processes but also highlights other aspects of the molecular dysfunctions that otherwise may have been overviewed. Next, some few examples are discussed to illustrate the utility of HD transcriptomics for the proposal of new research lines:

1. The relevance of intracellular signaling pathways. The first transcriptomics studies soon demonstrated impairment of signaling cascades, as in the case of cAMP/PKA-dependent signaling pathway. Thus, transcriptional dysregulation of diverse adenylyl cyclases, which promote cAMP synthesis, was concomitant to a reduction in the binding of their activator forskolin in HD mouse striata [22]. Moreover, stimulation of the cAMP pathway was beneficial for a HD cellular model [15], in which the activity of the downstream transcription factor cAMP-responsive element binding protein (CREB) was decreased. These observations have led to the study of CREB involvement in HD [42, 43] and to the use of phosphodiesterase inhibitors, which prevent degradation of cyclic nucleotides, as potential therapeutical means [44, 45]. The importance of disrupted intracellular signal transduction is further confirmed by the emergence of compensatory mechanisms to restrain the deleterious effects of altered cascades. This is the case for some G protein regulators (RASD family, member 2/Ras homolog enriched in striatum (Rasd2/Rhes), RAS guanyl-releasing protein 2/calcium and DAG-regulated guanine nucleotide exchange factor I (Rasgrp2/CalDEG-GEF1), and regulator of G protein signaling 2 (Rgs2)) which actively contribute to the mHtt-induced toxicity and are downregulated at the transcript level in HD (reviewed in Seredenina and Luthi-Carter [41]). Further inhibition of this type of signaling components may constitute promising therapeutical strategies.
2. Impairment of cholesterol homeostasis. Research investigating the involvement of cholesterol in HD was boosted by a microarray study of a HD-inducible striatal cell line in which genes related to lipid metabolism and cholesterol biosynthesis were found to be downregulated [31]. Thereafter, imbalance in the levels of cholesterol and related metabolites has been found in different HD models and in patients, proportional to the length of CAG expansion. This imbalance has been documented as a decrease in brain, plasma, or cultured fibroblasts, although with some examples of cholesterol accumulation (reviewed in Valenza and Cattaneo [46] and Leoni and Caccia [47]). Brain cholesterol is primarily found as a constituent of the myelin, followed by astrocytes and neurons, and participates in myelination, neurite outgrowth promotion, formation and maintenance of the synapses, integrity of the membrane microenvironment for neuronal channels and receptors, and vesicle transport and exocytosis. Therefore, impairment of the cholesterol homeostasis may have a relevant impact in neuronal signal transduction and vesicle trafficking in HD.

### Transcriptional Dysregulation Is Not Exclusive to the Brain

HD mouse models in which the mHtt transgene was designed to reproduce the ubiquitous expression pattern of the endogenous Htt [48] show altered transcriptomes in the retina [26] and in nonneuronal tissues, such as blood [49] and muscle [23, 25], in the latter case with apparently little contributions of diabetes and weight loss. Peripheral tissues facilitate access to biomaterial and open the possibility of their use in the identification of biomarkers. Transcriptomics can determine in an unbiased manner the expression changes associated with the disease in blood samples [19, 50–52] and skin fibroblasts from patients [53]. Indeed, late and early presymptomatic stages have distinctive profiles [51], further supporting the suitability of peripheral expression changes for measuring pathology progression.

### HD Transcriptional Signatures Are a Complex Mixture of Common and Specific Changes

To date, the best attempt to meta-analyze striatal profiles from different animal models (including knock-in and transgenic mice for different mHtt lengths) within the same study [38] revealed a high degree of concordance, although a definitive description of a consensus core HD signature is still lacking. Importantly, animal models are able to recapitulate relevant features of the transcriptional dysregulation associated with HD in patients [27, 36, 38, 54–57]. Common changes were also observed among different brain areas and in nonneuronal tissues [18, 23, 25, 28, 36] and more strikingly among different polyQ disorders (see “Final Remarks” section), despite the apparent overrepresentation of regional and model-specific changes.

The presence of specific changes is the result of several factors as discussed below:

1. Intrinsic properties of the tissues, such as sensitivity to degeneration and tissular-specific transcription. The striatum exhibits a special sensitivity to neurodegeneration, as demonstrated by general insults that severely affect striatal cells, such as hypoxia, hypoglycemia, and ischemia [58], systemic administration of neurotoxins (e.g., 3-nitropropionic acid (3-NP) and 1-methyl-4-phenyl-1,2,3,6-tetrahydropyridine (MPTP)) [59, 60], and somatic CAG instability of the Htt gene [61]. This latter susceptibility is correlated with the acceleration of the pathology and can be predicted at the transcriptome level in different tissues [62, 63]. Therefore, the striatum and, in particular, the caudate nucleus are the brain areas that show greater transcriptional dysregulation compared with cortical areas and the cerebellum, in accordance with the earliest and more severe manifestations of the disease in humans [36]. Moreover, in the striatal region, the profile may be pronouncedly distorted by a cell population shift (i.e., neuronal loss concomitant with glial activation). The effect of tissular-specific transcription is observed by the fact that striatal-enriched genes are generally more sensitive to dysregulation within the striatum [37] and also by the normal differential gene regulation across brain areas: genes with differential levels of expression tend to appear in the tissular-specific HD signature, whereas genes with similar expression levels tend to be part of nonspecific pathways that become altered in HD [30]. Whether this dichotomy is in concordance with specific and common transcriptional regulators remains to be fully determined.
2. Intrinsic properties of the models. For example, the promoter sequences driving transgene expression (e.g., ubiquitous vs neuronal-restricted) determine which cell types will be more affected, and the length of the mHtt determines the progression rate of the disease because N-terminal transgenes lack the required signals present in full-length mHtt (either transgenic or knock-in) to promote degradation in response to misfolding [64]. Gene profiles of fast and slow progressive models were initially described as not reproducible [65] mainly because they were retrieved from dissimilar stages of the pathology; once these models were comparable, the gene profiles were significantly similar [38]. The number of CAG repeats is also relevant, as initially suggested by transgenic models [33, 66] and as further confirmed in heterozygous knock-in systems with descriptions of a relatively consistent and prominent transcriptional signature associated with an increasing length of repeats [67, 68].
3. Analytical procedures. The intrinsic properties discussed above make performing a reliable meta-analysis of distinct gene profiles difficult; this limitation is more evident if based on the results of conventional pair-wise comparisons between the control and the aberrant Htt expansion situations using arbitrary thresholds. Exploring alternative approaches has led to interesting insights that otherwise would have remained undetected: for example, the aforementioned accumulative dysregulation associated with CAG repeats, the detection of subthreshold HD signatures in a less affected brain area [30], and the dissection of the relative contribution of cell populations to the HD transcriptional signature in heterogeneous tissues [69]. In the case of biomarkers, investigation of a natural human population characterized by confounding demographic and individual factors is challenging from an analytical point of view. A first study reported 12 promising candidates from the blood of human patients [51], which were not confirmed by other studies [50, 52], even with the reanalysis of the original data [70]. However, the collection of proposed biomarkers in these studies were to some extent reproduced in independent cohorts, indicating that integrative analytical procedures are required to reach a reliable consensus.

### HD Is Not Fully Explained by Mitochondrial Dysfunction

Mitochondrial dysfunction is supported by several pieces of evidence: the mitochondrial localization of mHtt, the defective Ca^2+^ homeostasis, the impairment of mitochondrial respiration and trafficking in mHtt-expressing cells, and the reduced activity of peroxisome proliferator-activated receptor gamma coactivator-1α (PGC-1α), which is relevant for the expression of genes related to mitochondrial biogenesis and oxidative phosphorylation [71]. 3-Nitropropionic acid (3-NP) irreversibly inhibits succinate dehydrogenase, the enzymatic activity required for the mitochondrial electron transport chain, and induces striatal neuronal degeneration and a HD-resembling phenotype [60]; thus, 3-NP treatment is regarded as a chemical-inducible model of HD. However, striatal gene profiling demonstrated a lack of correlation between mHtt-expressing and 3-NP-treated animals [18, 55] and immortalized cell lines [72]. This is in agreement with the comparison with PGC-1α knockout profile (see “PGC-1α” section). Overall, energy deficiency in HD is not fully explained by mitochondrial dysfunction, and extramitochondrial mechanisms should be investigated.

## Multiple Gene Expression Regulators Are Altered in Huntington’s Disease

Among the gene expression regulators, we can find transcription factors and associated cofactors, including chromatin-remodeling proteins and noncoding RNAs (ncRNA). Chromatin-remodeling proteins are gaining increasing interest in neuropathology as they are responsible for the epigenetic deficits observed in mental and neurological disorders [73, 74]. Research in HD and other polyQ disorders introduced epigenetics and epigenetic-based correctives early in the last decade, and these studies were soon extended to more common neurodegenerative disorders, such as AD. This led to the general hypothesis of epigenetic imbalance as one of the most important causes of the altered expression of genes involved in neuronal function and survival in neurodegenerative processes [75]. Since then, several modifications of histones (acetylation, methylation, ubiquitylation, and phosphorylation) and DNA (methylation and hydroxymethylation) have been described to be affected in HD (for further details, see Valor and Guiretti [76]). Regarding ncRNAs, microRNAs (miRNAs) have been extensively examined in the context of this polyQ pathology and in other neurodegenerative disorders [77]. miRNAs exert posttranscriptional inhibition by halting translation or by promoting RNA degradation [78], in the latter case consequently modulating the net transcript level of a given gene. As mentioned previously, several miRNAs are deregulated in HD, and in some cases, their potential target transcripts are found to be changed in the opposite direction [13, 14, 79]. Very recently, the involvement of a new class of ncRNAs, small CAG-repeated RNAs (sCAG-RNAs), has been described in HD. sCAG-RNAs are derived from the mHtt transcript and promote the degradation of messenger RNAs (mRNAs) containing CUG-rich sequences, with deleterious consequences [80], thus demonstrating that both mHtt protein and RNA can contribute to HD.

### Mechanisms for Disruption of Gene Expression Regulators

The activity of gene expression regulators can be affected in several ways, as defined in single-gene studies:

1. By a loss of interaction with wild-type Htt. The best-known example is the RE1-silencing transcription factor/neuron-restrictive silencer factor (REST/NRSF) that is physiologically retained by wild-type Htt in the cytoplasm. Thus, loss of a single allele promotes the nuclear translocation of this factor and alters the expression of neuronal genes [81], as discussed in the next section. Other examples are nuclear factor kappa B (NF-κB), which is translocated from dendrites to the nucleus through the interaction with wild-type Htt [82], and nuclear receptors [83], including liver X receptor (LXR), a key regulator of cholesterol metabolism [46, 47].
2. By aberrant interactions with mHtt. This is the best-characterized action and was originally proposed because several transcription regulators, such as the acetyltransferase CREB-binding protein (CBP), the histone deacetylase complex subunit mSin3a, the tumor suppressor p53, the transcription elongation regulator 1 (Tcerg1/CA150), and the TATA-binding protein (TBP), among many others [84–88], were found within the mHtt aggregates, suggesting a depletion of regulatory factors needed to maintain normal levels of expression. Other evidences further support mHtt-dependent transcription failure. For example, the expression of exogenous mHtt produces cell-autonomous transcriptional dysregulation, as documented by transcriptomics studies [12, 15, 33, 34], and alters in vitro reporter expression [15, 84, 89–91]. Additionally, there is a CAG repeat-dependent transcriptional response in the context of both loss- and gain-of-function, as previously mentioned. However, the pathogenic role of mHtt aggregates is still under debate, and recent evidence favors soluble mHtt as the toxic form responsible for the molecular failures observed in the pathology [92–95]. Regarding transcription, aggregative inclusions of mHtt did not significantly decrease or change the nuclear distribution of some transcriptional regulators [90, 96, 97], and soluble forms of mHtt were able to inhibit their activities per se [98–101], as occurs in the case of the housekeeping specificity protein 1 (Sp1), CBP, and TBP. In agreement with these observations, altered gene expression can appear prior to nuclear Htt aggregation as already discussed [31, 100, 102]. Finally, interference by mHtt can also occur at the promoter level through direct association with DNA [103, 104].
3. By the disruption of upstream gene expression regulators. Apart from miRNAs, transcription factors and associated cofactors can also be prominently deregulated at the transcript level, as retrieved by functional enrichment analyses of pathological gene profiles. Nonetheless, such dysregulation does not always contribute to the progression of the disease by affecting downstream target genes, but it can also be a part of the homeostatic response in an attempt to restore lost activities. An example is the CCAAT-binding protein nuclear factor Y (NF-Y) that is present in mHtt aggregates with a concomitant reduction in protein levels but upregulated at the mRNA level [105].
4. By abnormal protein degradation, by either promoting or preventing the removal of regulators such as CBP [76] or the Wnt-dependent transcriptional coactivator β-catenin [106], respectively.

### Genome-Wide Approaches to Link Transcriptional Dysregulation and Altered Regulator Activities

To gain further insight on the regulatory mechanisms responsible for HD signatures, gene expression datasets can be analyzed for the enrichment of transcription factor binding sites (TFBS) to infer the most promising factors involved in the transcriptional deficits. Along the same lines, the 3′UTRs of deregulated genes can be scanned for the putative binding of miRNAs. Early attempts, although limited by the scarce knowledge of genomic sequences at the time, were able to identify a small subset of deregulated genes with DNA motifs at their promoters for Sp1 [23] and CREB [15], the latter being implicated in neurodegenerative disorders [107]. Some recent reports have predicted the enrichment of more representative binding motifs in large datasets, such as the sequences for NF-κB, Sp1, p53, REST, the procaspase activator HIP1 protein interactor (HIPPI), or miR-22 [14, 16, 20, 21, 28, 72, 108, 109], to cite some examples.

There are, however, obvious limitations to the prediction strategy. First, regulatory proteins are able to bind through noncanonical consensus sequences or indirectly through the recruitment of other associated factors; second, the use of predicted DNA motifs may depend on the tissue-specific context, i.e., on the presence of specific cofactors and interacting partners; and third, this strategy is not valid for chromatin-remodeling proteins, as they do not recognize any primary sequence in the DNA. Finally, predictions are highly dependent on the assumptions made by the algorithms, which can be especially problematic in the case of miRNA-binding sequences. Two different approaches circumvent these issues: (i) meta-analysis of gene profiles to determine the degree of similarity between the HD signature and the transcriptional changes caused by genetic manipulation of particular regulators [66, 104] and (ii) mapping the genomic occupancy of nuclear proteins. This second approach offers an important advantage over the former one: both direct and indirect targets are distinguishable, without the confounding homeostatic response that may be highly specific for a particular genetically engineered model. In the HD field, initial efforts exploited array platforms to examine the DNA positions originally bound to the protein of interest after immunoprecipitation with a specific antibody in the so-called ChIP-on-chip approach [103, 110, 111]. As occurs with transcriptomics-based techniques, this approach was not fully unbiased but restricted to the probes spotted in the arrays, and usually the promoter regions of known genes were interrogated without considering the regulatory regions distal to the transcription start site (TSS), thus excluding enhancers and the promoters of undiscovered genes and alternative isoforms from the analysis. Currently, next-generation sequencing is the preferred approach to investigate genomic occupancy (ChIP-seq) [20, 21, 28, 66, 112, 113], as it enables nearly nucleosome resolution, allows the redefinition of the consensus DNA motif and possible variants, and explores the contribution of neighboring sequences.

Next, studies designed to link transcriptomics with the activity of particular expression regulators are discussed in detail.

#### REST

REST is a Kruppel-type zinc finger protein that was originally described as a repressor of neuronal genes in nonneuronal cells for maintaining the phenotypic identity of these cells, according to its high expression level in embryonic and nonneuronal cells, opposed to the lower level observed in neurons (reviewed in Adachi and Monteggia [114]). As previously commented, this repressor can enter the nucleus of HD neurons and repress the expression of cortical transcripts, such as brain-derived neurotrophic factor (BDNF) [81]. To identify additional HD genes that are dysregulated because of REST binding, chromatin lysates from patients with advanced grade of disease were examined by a ChIP-on-chip approach using an array specifically designed to explore canonical RE1/NRSE sites in the human genome [110]. To minimize the use of human material, it is possible to take advantage of the high functionality of REST in proliferative cells and employ ChIP-seq information derived from accessible cell lines to enlarge the list of candidate genes for further examination in HD signatures. In doing so, it was possible to identify the first long noncoding RNAs (expressed in the locus of human accelerated region 1 (HAR1)) as downregulated in the striatum of HD patients [115] and to tightly associate direct REST regulation with the decreased expression of miRNAs and neuronal genes in a HD cell model [116].

#### Forkhead Box Protein 1 (Foxp1)

Foxp1 and other members of the subfamily play important roles in cell-type-specific gene expression in the heart, lung, thymus, immune cells, and developing brain. Haploinsufficiency in humans may be the cause of an intellectual disability that severely affects the onset of speech and language and is also commonly associated with distinctive facial features, autistic traits, and congenital malformations [117]. Foxp1 shows high levels of expression in the striatum and is downregulated in HD models and in the caudate nucleus from patients [37]. Genome-wide analysis of the involvement of Foxp1 in HD transcriptional dysregulation has been conducted in a series of concatenated experiments. First, combined microarray and ChIP-seq analysis in a striatal cell line overexpressing this transcription factor identified a set of *bona fide* target genes, including some associated with inflammatory and immunological disorders in other systems [66]. In agreement with the in vitro results, viral transduction of Foxp1 primarily led to the repression of immune-related genes in the adult striatum. This pattern moderately anticorrelated with published HD transcriptional signatures of animal models and patients [66]. Although this study suggested an interaction with mHtt, it is not clear whether the Foxp1 actions in glial cells are direct, because the main transduced cells in the in vivo experiments were neurons, and endogenous Foxp1 binding has not been examined in a symptomatic scenario. In any case, modulation of Foxp1 activity offers a potential new strategy to ameliorate the polyQ pathology by restricting astrocytic and microglial activation in HD.

#### Heat Shock Factor 1 (HSF1)

HSF1 is a leucine zipper transcription factor and a key regulator of the heat shock proteins (HSPs), including chaperones involved in protein homeostasis (folding, degradation, targeting, etc.), the expression of which is induced in response to different types of stress. In fact, some reports demonstrated an altered gene expression pattern for some of these proteins [32, 105, 118], and ameliorative strategies aimed at enhancing either HSP or HSF1 activities have been proposed to be beneficial in HD (see “Transcriptomics-Based Ameliorative Strategies” section). To understand the potential role of HSF1 in HD, a ChIP-seq analysis was conducted in the mHtt-expressing immortalized striatal cell line ST*Hdh*Q111/Q111 [112]. In the basal state, relatively few loci were bound by HSF1 in this cell line, and the majority of these loci (~75 %) were the same as in the control ST*Hdh*Q7/Q7 cells. However, under heat shock conditions, HSF1 showed increased genomic binding in the control cell line that was impaired under the expression of the expanded polyQ version and affected more than one third of HSF1-dependent genes. Whereas proteostatic genes were enriched in the HSF1-associated signature in the two cell lines, genes showing deficits in both gene expression and HSF1 binding in the mutant condition were represented by other unrelated functions, suggesting that the response to HSF1-induced stress is largely preserved in this HD model. However, this situation is more complex in vivo [119], as we will see in brief. In any case, a significant number of HSF1 targets were found in the HD signature of animal models and patients, pinpointing the potential relevance of HSF1-dependent transcription in the pathology [112].

#### PGC-1α

PGC-1α is a transcriptional coactivator that regulates the expression of mitochondrial genes among other targets. Its potential relevance in HD has been suggested by a specific decrease in PGC-1α in the postmortem caudate nucleus of HD patients, a similar motor phenotype in PGC-1α knockout and HD mice that is worsened when combining both mutations and by the fact that overexpression experiments restoring PGC-1α levels ameliorated neurodegeneration and mHtt aggregation [104, 120]. In the seminal study by Krainc and coworkers [104], microarray analysis of knockout mice revealed deregulated genes involved in oxidative phosphorylation, mitochondrial function, and the electron transport chain. These pathways were also altered in HD patient profiles [36]. However, a deeper comparison with HD datasets did not show a significant transcriptome-wide overlap [55, 120], indicating that disruptions of mitochondrial pathways are not sufficient to elicit the widespread transcriptional response reported in HD, in agreement with the observation made with chemically induced mitochondrial dysfunction (see “HD Is Not Fully Explained by Mitochondrial Dysfunction” section).

#### Epigenetic Marks and Associated Proteins

Epigenetic marks that are linked to physiological processes, such as memory formation, and to intellectual disabilities and aging [121–123] have been investigated in a genome-wide manner in HD. These marks include those that are associated with active genes (H3K9/14 ac and H4K12ac in the promoters and adjacent regions of the R6/2 striatum [111] and in the nonrepetitive genome of the N171-82Q hippocampus [28] and H3K4me3 in the R6/2 cortex and striatum [21]) and with repressed genes (H3K9me3 [113] and DNA methylation [20] in ST*Hdh*Q111/Q111 and control ST*Hdh*Q7/Q7 cells). However, in the latter case, 5-methylcytosine (5-mC) and 5-hydroxymethylcytosine (5-hmC) were in principle not distinguishable. 5-mhC is highly enriched in neurons and may constitute an intermediate step in demethylation, leading to gene derepression [76]. The genomic pattern of this modification has been determined and shows a general reduction in the presymptomatic cortex and striatum of YAC128 mice [124]. Interestingly, changes in the local levels of 5-hmC correspond to changes in expression in the same direction for selected genes, suggesting a positive association with gene activation that is altered in HD.

Surprisingly, the genome-wide correlation between differential histone acetylation and gene expression is poor, despite observance of a net hypoacetylation [28, 111]. Methylation of either histones or DNA appears to be more correlated [20, 21, 113], most likely reflecting a distinct biological relevance for epigenetic marks in HD. Alternatively, it is tentative to speculate that the use of the same deep sequencing-based technology to map either gene expression or epigenetic marks may allow better data integration for comparative purposes. Even in these studies, the correlation is not globally consistent, as it is possible to find a high proportion of genes with no correlation or with a correlation opposite to expectations. At least three possibilities may explain these discrepancies: (i) additional regulatory mechanisms are required to totally connect transcriptional and epigenetic dysregulation (see “Toward an Integrative Model of HD Transcriptional Dysregulation” section), (ii) homeostatic responses may emerge in a locus-specific basis to eventually restore expression by alternative means, and (iii) epigenetic modifications may have differential roles or repercussions in transcript regulation depending on the genomic location. In this sense, a general theme emerges that the altered epigenetics at TSS and the surrounding regions are more prone to be correlated with altered expression in HD, thus affecting genes with primary roles in synaptic transmission, cytoskeleton-dependent processes, and signal transduction [20, 21, 28, 113]. In addition, broad islands of H3K4 methylation around TSS in control animals are better predictors than the sharp islands of downregulation observed in transgenic mice [21], indicating that the shape of the binding profile somehow reflects specialized functions in gene regulation.

To complement these results, a genome-wide landscape of chromatin-remodeling proteins is missing. At the moment, a reduction of the hypomethylation of the H3K4 mark in HD has been ascribed to the upregulation of the specific histone demethylase lysine (K)-specific demethylase 5C (KDM5C/SMCX/Jarid1c). This is supported by knockdown experiments that reported an increase in the expression levels of selected genes [21].

In the absence of nucleosome resolution, meta-analysis of transcriptome data from different studies may provide key insight. Histone deacetylation as a result of decreased acetyltransferase and increased deacetylase activities is present in several polyQ models, although this latter finding needs further clarification [76]. Anyway, general nonspecific deacetylase inhibition leads to phenotypical amelioration (see “Transcriptomics-Based Ameliorative Strategies” section). Therefore, identifying the relevant histone deacetylases (HDACs) in these beneficial effects may help in the development of more specific drugs. Studies in yeast revealed a significant overlap between mHtt-induced downregulation [125] and the target genes identified to bind Rpd3 (the ortholog of HDAC3) in a separate ChIP genomic mapping [126]. Interestingly, the yeast mutant for a component of the Rpd3 complex, Ume1, suppresses mHtt-induced toxicity [127]. In mice, the involvement of HDACs is more complicated, as the knockdown of several isoforms in a mHtt-expressing context did not have apparent beneficial effects [76]. The exception is HDAC4, the reduction of which improved a series of faulty phenotypical, electrophysiological, and biochemical properties without restoring the HD-associated transcriptome [128]. However, marginal changes such as the rescue of BDNF levels were observed. A parallel study of HDAC4 knockout postnatal brain casts doubt on the gene-repressive role of this protein, in contrast to the conclusions obtained in the overexpression approach [129], because the pattern of acetylation was not altered, and only minor changes were observed at the level of the transcriptome [130]. A strong functional redundancy by other family members is an alternative plausible explanation for these negative results.

#### Huntingtin

Interestingly, Htt can bind directly to DNA, as shown in immortalized striatal cell lines and in brain tissue from R6/2 mice and patients, not only at the promoter level but also to intronic and intergenic regions, including repetitive sequences [103]. Because the cell lines were derived from homozygous knock-in strains bearing different polyQ extensions, it was feasible to compare the behavior of two different Htt versions (bearing the normal and a pathological length of polyQ) at the DNA binding level. Strikingly, they exhibited a distinct pattern of genomic occupancy at the promoter level in a ChIP-on-chip approach, with no correlation between Htt occupancy and gene expression changes. Variants of recombinant Htt-exon 1 were able to bind in vitro to multiple known DNA motifs in a polyQ-dependent manner and change the conformation of the DNA [103], suggesting that the effects of Htt binding may involve a generic and most likely nonspecific mechanism of interference and alteration of chromatin structure. The function of wild-type Htt in the nucleus deserves further investigation, despite its low abundance in this compartment, as an early report suggested a scaffolding role for multiprotein complexes in nuclear processes [131].

### Toward an Integrative Model of HD Transcriptional Dysregulation

A review of the vast literature together with a systematic analysis of transcription factor activities as performed by Cha and colleagues [103] leads to the conclusion that dozens of gene expression regulators are affected in HD. Although the precise contribution of each regulator to the HD signature remains to be determined with temporal and regional precision, it is reasonable to conclude that a few factors cannot be responsible for the complex gene profiles observed in the transcriptomics studies. Causal relationships are difficult to tract, and homeostatic responses are intermixed with deleterious dysregulation [41]. Moreover, our still limited understanding of basic regulatory processes, such as those represented by epigenetic mechanisms in neuronal gene expression, enhances our handicap in determining the precise origin of HD transcriptional dysregulation ([76], Lopez-Atalaya and Barco, in preparation).

**Fig. 2:**
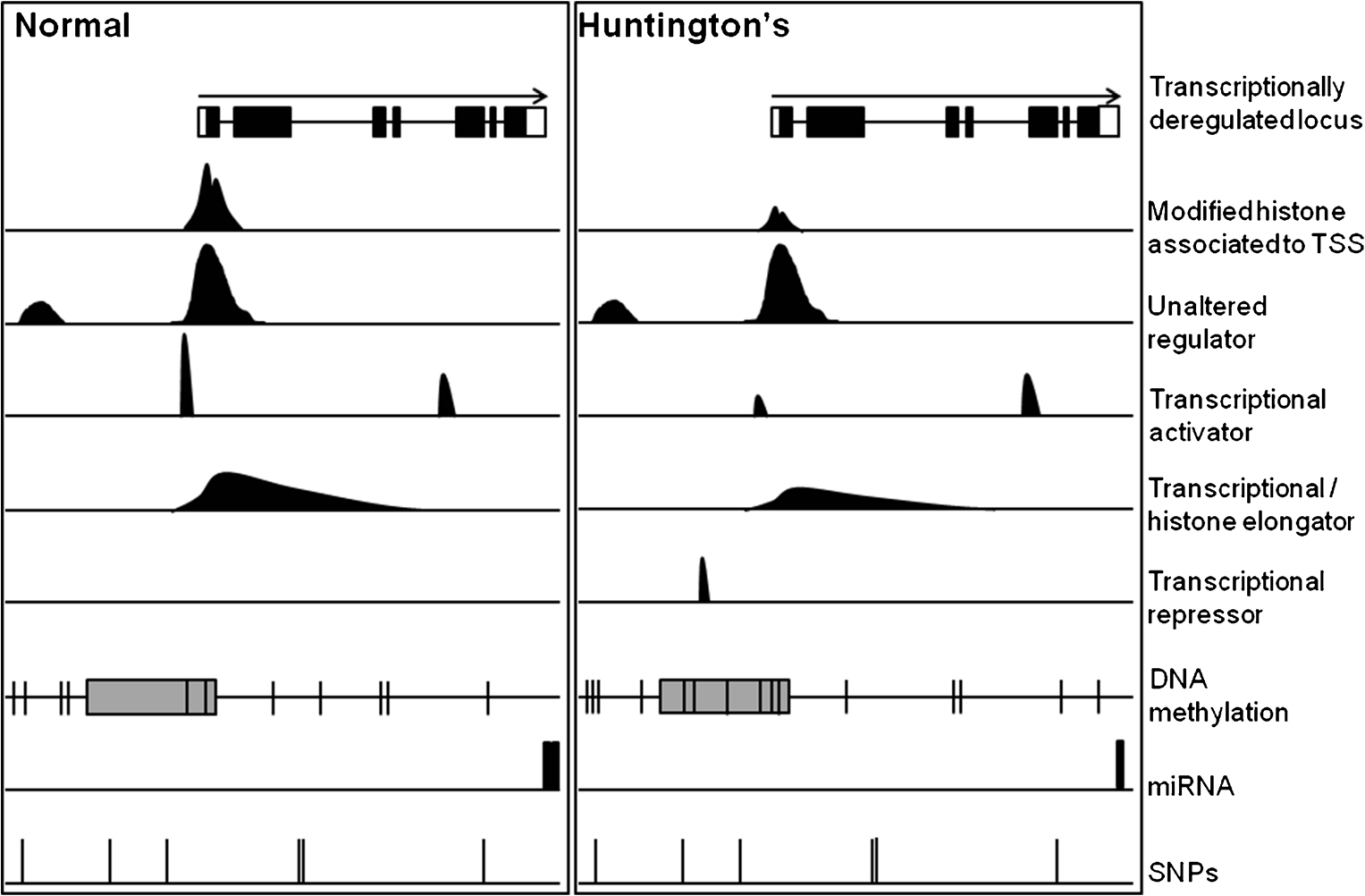
Convergent disruption of regulatory mechanisms for differentially expressed genes in HD. Schematic representation of a hypothetical deregulated gene in HD, which transcriptional dysregulation may be the result of correlated changes in the complex genomic redistribution and occupancy of transcription factors, epigenetic marks, and miRNAs. SNPs, although invariant, may contribute to the susceptibility to the expression change. *Arrow*, direction of the gene; *shade box*, CpG island

Integrating different regulatory mechanisms may provide a more complete picture, as changes in expression levels may be the result of disruption of convergent regulators (Fig. 2). Transcription factors may shape epigenetics through the control of chromatin-remodeling protein expression, as in the case of histone methylation. The reported global hypermethylation of H3K9 in HD mouse models and in patients [132–135] has been explained as a result of abnormal increase of binding activity to the promoters of chromatin regulator genes: (i) the binding of Sp1 and Sp3 to the promoter of the histone-lysine N-methyltransferase SET (SU(VAR)3-9, enhancer of Zeste, Trithorax) domain bifurcated 1 (SETDB1, also known as ESET) [134] and (ii) the binding of caudal type homeobox 2 (Cdx2) to the promoter of the DNA-dependent ATPase/helicase alpha thalassemia/mental retardation X-linked (ATRX) [136]. Interestingly, it has been proposed that CBP is an indirect repressor of SETDB1 [137], which reduction in HD may also contribute to SETB1 upregulation. In another example, REST appears to be a primary cause of miRNA dysregulation in HD [138] that can also influence epigenetics. In combination with the corepressors Sin3a/b and CoREST, REST recruits multiple chromatin-remodeling complexes containing histone methylases (G9a) and demethylases (LSD1 and KDM5C), histone deacetylases (HDAC1 and HDAC2), and the methyl–CpG-binding protein MeCP2, among others. REST binding moderately correlates with decreased histone posttranslational modifications associated with active genes and increased histone methylation associated with repression [138]. To be more precise, a decoy oligodeoxynucleotide containing REST motifs was able to remove REST from the promoters of downregulated genes in mHtt-expressing striatal cell lines, with an increase in K9H3ac levels and a reversal of expression deficits [139]. Finally, miR-9/miR-9* closes the circle by providing a feedback mechanism for its own REST-dependent transcriptional decrease in HD as it regulates REST and CoREST expression [13]. Although causality has not yet been well established, the relationship between transcription factors and epigenetics has been assessed at the genomic scale. In particular, altered patterns of DNA methylation have been linked to the binding of the transcription factor Fos-related antigen 2 (Fra-2), the proto-oncogene JunD, and sex-determining region Y-box 2 (Sox2) [20]. Bisulfite sequencing of the DNA from the ST*Hdh*Q111/Q111 and ST*Hdh*Q7/7 cell lines identified differential patterns of cytosine methylation that were more evident at CpG-poor regions. The primary focus was on low methylated CpG-poor regions, as they could be potentially created by transcription factor binding, as reported in embryonic stem cells [140]. While seeking candidates, Sox2 and the AP-1 members Fra-2 and JunD were further investigated, as they were deregulated at the transcriptional level, and their binding motifs were enriched in these regions. Confirming expectations, ChIP-seq experiments showed a complete loss of Sox2 in mHtt-expressing cells and a negative correlation between changes in the binding of AP-1 factors and DNA methylation, primarily in distal positions [20].

Another aspect to consider for integration is the fact that histones and transcription factors can be posttranslationally modified by the same enzymes. For example, mitogen- and stress-activated kinase 1 (MSK-1) phosphorylates both histone H3 and CREB [141], and CBP acetylates not only histone tails but also transcription factors relevant to HD, such as p53. The tumor suppressor factor p53 interacts with mHtt [84] and can mediate the cytotoxicity and mitochondrial dysfunction initiated by mHtt [142]. More recently, p53 was found to be hyperacetylated in HD models, in accordance with a proapoptotic role [143], although mHtt expression could also induce hypoacetylation at the same residue [144]. These apparently paradoxical results can be explained by the complex pattern of p53 acetylation [145] because different combinations of lysines become acetylated in response to apoptosis and DNA damage or to the neuroprotective effect triggered by pharmacological treatment [146]. Another example of CBP-target protein is the RNA polymerase I factor upstream binding factor 1 (UBF1), found to be depleted in both animal and cellular HD models together with downstream ribosomal precursor mRNA [147]. Overall, due to its multiple interactions, CBP may act as a scaffolding and acetylation source for several nuclear proteins within the same locus.

## Transcriptomics-Based Ameliorative Strategies

In addition to elucidating the cause and consequences of transcriptional dysregulation in HD, transcriptomics analysis has been used to test the efficacy of ameliorative strategies that directly or indirectly restore the altered activity of gene expression regulators. In this section, the genome-wide studies aimed at analyzing the transcriptional changes associated with amelioration by genetic and pharmacological means are summarized.

### Gene Profiling Analysis for the Design of Ameliorative Strategies

Transcriptional profiles can be considered to be intermediate outcomes between the origin of the pathology (e.g., mutation of the Htt gene in HD) and the phenotypical manifestation at the level of behavior. Brain transcriptomes have the potential to record experiences, plasticity events, perturbation episodes, and medical interventions either in a transient or, as often occurs with neuropathologies, in a permanent manner. In the case of HD, the accumulation of gene expression rearrangements involves direct regulatory mechanisms, cytosolic processes that affect the fine-tuned activation of downstream signaling pathways converging in the nucleus, and the transcriptional homeostatic responses to minimize damage, which together constitute the “disease signature.” In the same way, it is possible to define the “ameliorative signature” as the fraction of genes which expression is restored by a particular treatment [148, 149]. This signature is also useful in the search for biomarkers for monitoring progression deceleration. In theory, the analysis of HD transcriptomics can (i) provide a quantifiable output of the suitability of preclinical therapeutics, (ii) propose novel strategies based on meta-analyses of concurrent genome-wide data, and (iii) identify the players involved in amelioration, to finally obtain invaluable insights for the improvement and design of ameliorative strategies.

#### Evaluation of mHtt Targeting

Long-term silencing of mHtt by RNA interference (based on either small hairpin RNA or artificial miRNAs) represents a promising strategy to alleviate the gain-of-function effects in patients [150]. To reach an optimal balance between efficacy and safety, the effect of nonallele-specific interfering RNAs has been studied. In two different designs [151, 152], nonspecific targeting of Htt proved to be beneficial in vivo despite knocking down the endogenous wild-type variant; tolerance to wild-type Htt silencing is not exclusive to rodents and has also been described in adult rhesus macaques [153]. Of note, there were transcriptional changes caused by the delivery of these interfering RNAs in the control striatum. This transcriptomic effect, associated with wild-type Htt reduction, was partially consistent with diverse knockdown scenarios in HD models [36, 38, 154, 155], thus confirming the loss-of-function signature in HD. A third study minimized the toxic side effects of off-targeting silencing (unintended silencing of unrelated genes) by focusing the design of interference RNAs on the seed sequence that recognizes the Htt transcript. A screening complemented with microarray analysis in transiently transfected HEK293 cells identified two small RNA candidates that met the criteria of minimal off-targeting (very low number of altered transcripts) and high efficacy (large reduction in the Htt transcript). Subsequent long-term assessments demonstrated a lack of overt neurotoxicity in vivo [156].

#### Modulators of polyQ-Induced Toxicity

One of the main advantages of proliferative cellular-based models is the ability to conduct large systematic and unbiased screenings. Regarding mHtt-expressing cells, this approach allows the identification of both tractable intracellular pathways and novel compounds by investigating the reversal of polyQ-induced cell death. This information would be useful for the posterior design of therapeutics in animal models. Target candidates for amelioration can be determined within the HD signature. Thus, overexpression may potentially restore cellular deficits (e.g., glucose metabolism and oxidative stress) [12] and potentiate the so-called homeostatic response; for example, a significant proportion of the upregulated genes in stably transfected cell lines were able to promote the clearance of mHtt and slow its accumulation in subsequent overexpression experiments [157]. Alternatively, the screening of a library of compounds allows for the proposal of mechanisms of death prevention by using transcriptomics information as an unbiased starting point. This is the case of the microtubule destabilizer podophyllotoxin, which enhances survival through the activation of the ERK pathway via the microtubule-associated Rho/Rac activator, guanine nucleotide exchange factor H1 (ARHGEF2/GEF-H1) [158].

#### Combined Pharmacological Treatments

Cocktail preparations may have synergic actions in improving cognitive and motor symptoms. This is the basis for the triple treatment used in the fast progressive R6/2 transgenic model. This treatment aims to increase neurotransmitter levels and mitigate mitochondrial dysfunction simultaneously, using the acetylcholine esterase inhibitor tacrine, the monoamine oxidase A inhibitor moclobemide, and creatine [159]. Interestingly, this combined treatment alleviates the HD phenotype more successfully than single-drug administration and globally reverses transcriptional changes: nearly 50 % of the profile returned to normal levels.

#### Environmental Enrichment

Several reports indicated that the housing conditions of HD mice have an impact on disease progression and opened the possibility for occupational-related therapies. Thus, animals maintained under environmental enrichment (i.e., with opportunities for frequent exploration, interaction with novel objects, climbing, and other voluntary exercise in a changing environment) exhibited phenotypical amelioration and an extended survival rate [160–163]. Although long-term environmental enrichment is known to rescue deficits in genes, such as BDNF, the cannabinoid receptor 1, and the striatal marker dopamine and cAMP-regulated neuronal phosphoprotein (DARPP-32/Ppp1r1b)) [164], a global transcriptomic analysis failed to fully associate the effects of enrichment with any change in gene expression and concluded that the beneficial effects were due to a reduction in aggregate load [165]. In contrast, the environmental enrichment paradigm was reported to cause significant transcriptional rearrangement in other studies [166–168], probably indicating the need for standard procedures.

#### Restoration of BDNF Levels

The meta-analysis of datasets from diverse sources, as discussed in the “Genome-Wide Approaches to Link Transcriptional Dysregulation and Altered Regulator Activities” section, can be extended to other gene products not directly related to expression regulation. In cases where the overlap is significant, it may be feasible to investigate the ability of available drugs to modulate newly identified targets or their associated pathways. This principle is well represented by the prosurvival factor BDNF. Depletion of wild-type Htt not only leads to the transcriptional repression of the *Bdnf* locus but also interferes with the trafficking of BDNF-containing vesicles. Overall, cortical supply of this neurotrophic factor to the striatum becomes impaired in HD [169]. Cross-comparison between HD and BDNF knockout datasets from the striatum revealed their high similarity, indicating the importance of BDNF deficits in this pathology. However, the concordance of BDNF-deficient profiles was higher in HD caudate patients compared with the transgenic R6/2 model, in agreement with a primary loss-of-function mechanism to alter BDNF levels. In any case, the ameliorative potential of BDNF-based therapies was demonstrated in HD animal models [170, 171], although a corresponding reversal of the HD transcriptome remains to be tested.

### Targeting Expression Regulators

Because transcriptional dysregulation is an early event in HD, directly targeting expression regulators may have an influence on the disruption of other processes in the neuron. This idea is supported by numerous experiments that rescued altered transcriptional activities by either overexpressing activators or knocking down repressors, thus restoring the expression levels of selected target genes and improving the well-being of sick animals and unhealthy cells. Interestingly, augmentation of the expression levels of wild-type Htt at the transcriptional regulatory level has been proposed to restore its physiological roles, as exemplified by homeodomain transcription factor Engrailed, which prevents mHtt aggregation and neurodegeneration by activation of endogenous Htt in a *Drosophila* model of HD [172]. The next section summarizes ameliorative strategies that have been tested in HD cellular and animal models to correct aberrant nuclear activities and have been monitored by gene expression profiling.

#### HDAC Inhibitors (HDACi)

The drugs belonging to different chemical families that are capable of inhibiting deacetylase activity are generally referred to as HDACi. Therefore, these drugs counterbalance possible deficits in acetyltransferase activity and restore the acetylation levels of histones and other nuclear proteins. This is the main proposed mechanism underlying the transcriptional-dependent amelioration of pathophenotypical traits observed in HD models (see Valor and Guiretti [76] for further details). In an attempt to identify the target genes responsible for such amelioration in HD, Ferrante and coworkers conducted a series of microarray studies in mouse models chronically treated with butyrate compounds. The correction of the HD signature was extremely limited [135, 173]; however this correction can involve a few highly relevant candidates for the mouse, including proapoptotic caspase-9 [135]. Alternatively, HDACi can exert their effects through the indirect activation of compensatory mechanisms. This is the case of the inhibition of the cytosolic sirtuin 2 (Sirt2), which failed to globally correct transcriptional dysregulation but reduced the shuttling of sterol regulatory element binding protein 2 (SREBP-2) to the nucleus, promoting downregulation of sterol biosynthesis genes independently of the mHtt expression. Thus, Sirt2 inhibition conferred neuroprotection by modulating cholesterol homeostasis [174]. In terms of HD signature reversal, the use of HDACi 4b was more successful. This treatment definitively normalized the expression of one third of deregulated genes, with a total trend toward normalization of more than 80 % of altered genes in the cortex, striatum, and cerebellum in transgenic R6/2 mice [56]. Importantly, HDACi treatment may have an impact, although subtle, on peripheral biomarkers [50, 51] that can be monitored for amelioration during treatments. It is worth noting that the acetylated status of hundreds of proteins can be influenced by HDAC inhibition [175]. Therefore ameliorative strategies based on the use of HDACis are beneficial due to their combined effects on histone and nonhistone substrates, either nuclear or cytosolic.

#### Anthracyclines

These drugs with antibiotic and anticancer properties have different mechanisms of action that involve interaction with the DNA, including histone eviction [176]. In the case of the two anthracyclines tested in pharmacological therapy of HD, mithramycin and chromomycin, binding to the minor groove of DNA promotes the displacement of GC-rich binding regulators, such as the Sp1 family members, with important consequences on restoring the imbalance between histone methylation and acetylation [76]. Biochemical and phenotypical amelioration was accompanied by the correction of a relatively small number of genes, including DARPP-32 and calcium/calmodulin-dependent protein kinase Camk1g, as determined by microarray analysis [133].

#### Activators of Heat Shock Proteins (HSPs)

Due to the protective actions of HSPs in the management of misfolded and aggregated proteins, it is not surprising that ameliorative strategies aimed at enhancing HSP activity reduced polyQ-induced toxicity in parallel with intracellular mHtt aggregate load. This enhancement can be achieved by using chemical inhibitors of the ATP binding of HSP90, including the antibiotic geldanamycin and its derivatives, 17-AAG and 17-DMAG, thus affecting the repressive actions on HSF1 expression, the master regulator of HSPs [177]. Bates and colleagues explored the beneficial effects of the HSP90 inhibitor NVP-HSP990 in the R6/2 model [119]. This study is an interesting example of dissecting the failure of a long-term ameliorative strategy to extract meaningful conclusions for further therapeutical design. Although this treatment demonstrated an improvement in the rotarod task performance and aggregate load reduction in the early stage of the pathology, this amelioration was later lost at the same time that the gene induction of HSPs was impaired, as observed by microarray analyses. This impairment was explained on the basis of a reduction in the binding of HSF1 at HSP gene promoters, where histone H4 was found to be hypoacetylated [119]. ChIP-seq analysis in a *Drosophila* cell line demonstrated that histone acetylation determines the inducible binding of the fly HSF1 ortholog [178]. Therefore, reversing histone acetylation deficits (e.g., by HDACi treatment) may extend the activation of HSPs in the treatment of HD.

#### REST Inhibitors

Screening libraries of compounds may identify novel drugs that modulate the activity of deregulated expression regulators in HD. In a structural-based approach, one screen retrieved quinolone-like compound 91, which reduced REST occupancy at the BDNF locus by reducing the activity of the REST-interacting partner Sin3b [179]. In another screen, several derivatives of benzoimidazole-5-carboxamide and pyrazole propionamide were able to reduce the luciferase activity driven by REST-binding motifs [180]. One of these derivatives (X5050) exerted its action by promoting REST protein degradation without interfering with its DNA binding or transcript levels. Confirmatory microarray analysis of neuronal stem cells treated with X5050 demonstrated a transcriptomic effect in which upregulation was predominantly enriched in neuronal genes predicted to be controlled by REST, with a concomitant downregulation of chromatin-related genes. As a validation of the activity of X5050 in a HD context, the expression of neuronal markers was increased after treatment in a stem cell model of HD and in chemically lesioned striata [180].

## Final Remarks

Aberrant expanded polyQ stretches, independently of the gene context, may affect similar regulatory mechanisms that should be reflected at the gene expression level. Thus, models for other polyQ disorders, such as dentatorubropallidoluysian atrophy (DRPLA), spinobulbar muscular atrophy (SBMA) or Kennedy disease, and spinocerebellar ataxia types 1 and 7 (SCA1, SCA7), share altered transcriptional genes with HD signatures [22, 26, 30, 181, 182].

Similarities can be extended to other neurodegenerative disorders that exhibit common characteristic hallmarks, such as channel-mediated cytotoxicity, mitochondrial dysfunction, intracellular trafficking, and deposition of misfolded proteins may influence and be influenced by shared transcriptional and epigenetic regulatory mechanisms. Therefore, it is not surprising that similar ameliorative strategies in HD are also applicable in other neuropathologies. For example, HDACi have been tested in cognitive pathologies, including neurodegenerative disorders and intellectual disabilities: HD, AD, Parkinson’s disease (PD), stroke, traumatic brain injury, amyotrophic lateral sclerosis (ALS), Friedrich ataxia, SBMA, DRLA, and Rubinstein-Taybi syndrome [76, 183, 184]. Anthracyclines have been used in ischemia and oxidative stress [185, 186], and induction of HSPs has been tested in neurodegenerative conditions with a notable presence of protein aggregation (AD, PD, ALS, Prion disease, etc.) [187]. Vice versa, useful insight for HD research can be obtained from transcriptomics analysis performed in models for other disorders, such as the effects of BDNF and environmental enrichment in AD [167, 188]. Although still very preliminary, meta-analyses of gene profiles from different neurodegenerative models have been conducted to identify common tractable pathways, such as the immune response and insulin-related pathways and synaptic-related functions [189–191] to put some examples. However, we need to learn more about HD transcriptional dysregulation, and analytical and experimental procedures should be standardized for better comparisons between conditions. For example, a refinement of the gene profiling studies of HD models at the temporal and cellular resolution is necessary. The use of LCM for the isolation of specific subpopulations, such as the cortical neurons of layer 5 [192] and Purkinje neurons [181], and the generation of animal models with highly specific expression patterns of the mHtt transgene [57], produce cellular-specific HD signatures with improved sensitivity over whole tissues. Techniques such as fluorescent-activated cell sorting (FACS) or translating ribosomal affinity purification (TRAP) can be useful alternatives. In any case, novel technologies, such as next-generation sequencing, are able to unravel unsuspected features (novel RNA species) or complex relationships (as occurs between transcriptional dysregulation and epigenetic marks). Moreover, the compilation and integration of genome-wide data, including transcriptomics, proteomics, epigenomics, and genomic occupancy, will be necessary for the full comprehension of the biological implications of transcriptional dysregulation in HD and to explore the full potential of genome-wide approaches in the design of improved therapeutics.
